# A Comprehensive Review of the Integration of Biotechnology in Modern Medicine and Pathology: Advances in Diagnostic Techniques, Therapeutic Innovations, and Clinical Outcomes

**DOI:** 10.7759/cureus.111463

**Published:** 2026-06-25

**Authors:** Santosh Jayant, Nithi Doley, N Swapna, Hari Prasad E, Kumar Sambhav, Baijnath Das

**Affiliations:** 1 Department of Pathology, Amaltas University, Bangar, IND; 2 Department of Pathology, Neelima Institute of Medical Sciences, Hyderabad, IND; 3 Department of Pathology, Aarupadai Veedu Medical College and Hospital, Vinayaka Mission’s Research Foundation (Deemed to be University), Kirumampakkam, IND; 4 Department of Microbiology, Sree Balaji Medical College, Bharath Institute of Higher Education and Research, Chennai, IND; 5 Department of Anatomy, All India Institute of Medical Sciences, Bilaspur, Bilaspur, IND; 6 Department of Medical Laboratory Technique, Teerthanker Mahaveer University, College of Paramedical Sciences, Moradabad, IND

**Keywords:** biotechnology, molecular diagnostics, nanomedicine, pathology, precision medicine

## Abstract

Biotechnology in modern medicine and pathology encompasses molecular diagnostics, multi-omics analysis, nanotechnology-enabled platforms, digital pathology, bioinformatics, and computational approaches that support disease detection, therapeutic development, and clinical decision-making. This review examines how these technologies have contributed to diagnostic workflows, therapeutic development, disease classification, and clinical decision-making in contemporary healthcare. The integration of molecular biology, nanotechnology, and computational sciences has expanded the evaluation of disease-related processes such as genetic variation, biomarker expression, tumor heterogeneity, immune regulation, and molecular pathway alterations in conditions including cancer, inherited disorders, infectious diseases, cardiovascular disease, and autoimmune disease. Therapeutic innovations such as gene editing, nanomedicine, immunotherapy, biologics, and targeted drug delivery systems have further supported mechanism-based treatment strategies by improving tissue targeting, reducing off-target toxicity, and enabling more individualized therapeutic planning. Artificial intelligence (AI) and bioinformatics approaches, including machine learning, deep learning, computational pathology, digital whole-slide image analysis, and omics-data integration, have supported biomarker discovery, disease classification, diagnostic image analysis, risk stratification, and pathology-based clinical decision support. Additionally, molecular and digital pathology have improved disease subclassification and prognostic assessment by integrating histomorphologic findings with molecular and computational data. Despite these advances, high costs, technical complexity, data standardization challenges, infrastructure limitations, and ethical concerns continue to restrict widespread clinical adoption. Future work should prioritize low-cost point-of-care molecular and biosensor platforms for resource-limited settings, interoperable standards for omics and digital pathology data, multicenter prospective validation of AI-assisted diagnostic tools and nanomedicine-based therapies, transparent reporting of algorithm provenance, and regulatory pathways addressing data privacy, clinical accountability, and equitable access. Overall, biotechnology represents an important component of precision healthcare, with the potential to strengthen diagnostic accuracy, targeted therapy, disease monitoring, and patient-centered clinical outcomes.

## Introduction and background

Biotechnology refers to the application of biological systems, biomolecules, cells, and engineering-based technologies to develop diagnostic, therapeutic, and research tools for medicine and pathology. The clinical need for biotechnology integration arises from the limitations of conventional approaches in explaining disease mechanisms, identifying molecular subtypes, monitoring treatment response, and supporting targeted therapy. In regenerative medicine, biotechnological advances have been reviewed in relation to endometrial disorders and tissue repair strategies [1]. Biotechnology-related bioactive compounds, including 5-hydroxytryptophan, have also been studied for their natural occurrence, biosynthesis, physiology, biotechnology applications, and toxicology [2]. Cellular and biochemical studies have examined mechanisms such as KLK8-mediated hippocampal neuronal apoptosis in stress-related experimental models [3]. Photodynamic therapy has been discussed as a biotechnology-related therapeutic strategy with biomedical applications [4].

Biotechnology also includes biomimetic delivery systems, molecular diagnostic tools, nanomedicine, imaging-guided sampling, and computational pathology. Macrophage cell membrane-based nanoparticles have been investigated for targeted delivery and treatment [5]. Mitochondria transfer and transplantation have been explored as biotechnological approaches with therapeutic potential [6]. Modern molecular diagnostic methods support the detection and interpretation of disease-associated molecular alterations [7]. Biomimetic materials have been evaluated in repair-focused biomedical applications, including enamel remineralization and repair [8]. Prokaryotic Argonautes have also been investigated as biotechnology-based molecular tools for in vivo biotechnology and molecular diagnostic applications [9].

Several biotechnology-related platforms support disease-specific diagnostic and therapeutic research. Nanomedicine has been explored for targeted atherosclerosis therapy through plaque-directed delivery, plaque clearance mechanisms, and inflammation modulation [10]. Galectin-carbohydrate interaction studies have contributed to understanding molecular recognition processes relevant to biomedicine and biotechnology [11]. Research on fusion RNAs has improved the understanding of oncogenic mechanisms and has potential diagnostic relevance in the molecular pathology of cancer [12]. In vitro culture systems have been used as alternative platforms for secondary metabolite production [13]. Optical imaging-guided targeted sampling has been investigated for precise diagnosis and molecular pathology assessment [14]. Biomimetic therapeutic approaches have been explored for inflammasome-related intervention in vascular dementia research [15]. Nanomedicine strategies have been investigated for autoimmune disease applications [16], anticancer applications [17], and thrombolytic therapy [18].

Computational and molecular pathology further illustrate the translational relevance of biotechnology. In computational pathology, specimen and data provenance are essential for reliable artificial intelligence (AI)-based diagnostic tools and clinical translation [19]. Molecular pathology has contributed to improved characterization of rare progeroid diseases [20]. Biotechnology-based tissue delivery approaches, including chenodeoxycholic acid-related pharmaceutical applications, have also been explored in preclinical settings [21]. However, existing discussions often describe these applications separately, without fully integrating their diagnostic, therapeutic, pathological, translational, and implementation-related implications.

Objectives of the review

This review aims to provide a clinically oriented synthesis of biotechnology applications in modern medicine and pathology, organized across major domains including molecular diagnostics, AI/bioinformatics, multi-omics, nanomedicine, targeted delivery, and regenerative or gene-editing approaches. It also distinguishes established clinical tools from emerging translational and experimental technologies, emphasizing clinical applicability, validation status, implementation barriers, and future research needs.

## Review

Methodology

Review Design

This comprehensive narrative review was conducted to summarize biotechnology applications in modern medicine and pathology, with an emphasis on diagnostic techniques, therapeutic innovations, computational pathology, precision medicine, and translational challenges.

Literature Search Strategy

A structured literature search was performed using PubMed, Google Scholar, and ScienceDirect for articles published up to June 2026. The search strategy used combinations of keywords and Boolean terms, including “biotechnology” AND “medicine,” “biotechnology” AND “pathology,” “molecular diagnostics,” “nanomedicine,” “digital pathology,” “artificial intelligence” AND “pathology,” “bioinformatics,” “multi-omics,” “liquid biopsy,” “precision medicine,” “targeted drug delivery,” “regenerative medicine,” “biosensors,” and “therapeutic innovation.” Reference lists of relevant articles were also manually screened.

Eligibility Criteria

Eligible articles included reviews, systematic reviews, original research articles, and high-impact studies addressing biotechnology-related diagnostic, therapeutic, pathological, precision medicine, or translational applications. Only English-language articles were included. Articles were excluded if they were duplicate records, unrelated to medicine or pathology, focused only on non-clinical industrial biotechnology, lacked diagnostic or therapeutic relevance, or did not provide sufficient scientific or clinical context for narrative synthesis.

Study Selection and Data Synthesis

**Figure 1:**
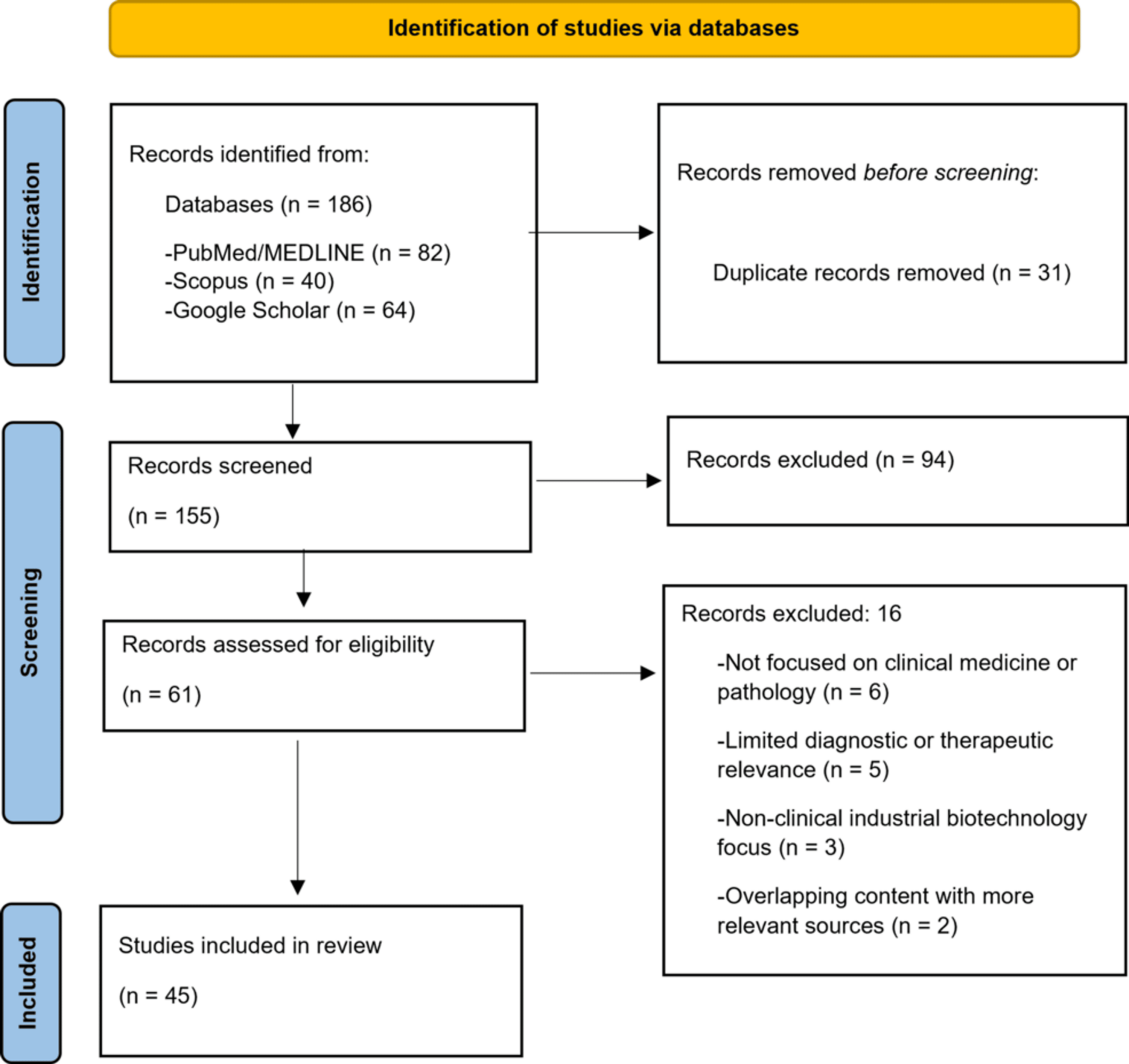
Preferred Reporting Items for Systematic Reviews and Meta-Analyses (PRISMA)-style flow diagram of literature selection.

Titles and abstracts were screened first, followed by full-text assessment of potentially relevant articles. Final inclusion was based on relevance to the review objectives, clinical or translational importance, and contribution to diagnostic, therapeutic, pathological, or implementation-related discussion. Evidence was narratively synthesized because the included studies differed in design, technology type, disease focus, clinical maturity, and reported outcomes. No meta-analysis or pooled statistical analysis was performed, and clinical claims were interpreted cautiously according to technology maturity, including established clinical tools, emerging translational technologies, and experimental approaches. A Preferred Reporting Items for Systematic Reviews and Meta-Analyses (PRISMA)-style flow diagram was prepared to summarize the literature identification, screening, eligibility assessment, and inclusion process. As shown in Figure 1, 186 records were identified, 155 records were screened after duplicate removal, 61 records were assessed for eligibility, and 45 studies were included in the final synthesis.

Biotechnology in medicine and pathology

Biotechnology in medicine and pathology includes disease-focused diagnostic, therapeutic, and analytical approaches, including immuno-oncology, biomimetic delivery systems, molecular pathology, and computational pathology. For clarity, these applications can be organized into major domains, including therapeutic biotechnology, biomimetic and targeted delivery platforms, biomolecular research tools, molecular pathology, and AI-supported computational pathology. Immuno-oncology illustrates one biotechnology-related therapeutic area in which immune-response modulation has been applied to cancer treatment [22]. Biomimetic nanoparticle platforms, including macrophage cell membrane-based nanoparticles, have been investigated for targeted delivery and treatment, showing how cell membrane engineering can be used in therapeutic development [5]. In vitro culture systems provide controlled experimental platforms for producing secondary metabolites and studying bioactive compounds with potential biomedical relevance [13]. Biotechnology applications in medicine also include experimental genetic manipulation, such as CRISPR-based methods used in stem cell lines for disease modeling and therapeutic research [23]. These examples differ in clinical maturity: immuno-oncology is clinically established for selected indications, whereas many biomimetic delivery systems and CRISPR-based stem-cell applications remain translational or experimental. Galectin-carbohydrate interaction studies have contributed to understanding molecular recognition processes relevant to biomedicine and biotechnology [11]. Cellular and biochemical disease research has also examined molecular mechanisms such as KLK8-mediated hippocampal neuronal apoptosis in experimental stress-related models [3]. Nanomedicine platforms have been investigated for disease-specific intervention, including dual-targeting strategies designed to regulate inflammatory injury and tissue damage in acute pancreatitis models [24].

Protein-protein interaction studies have strengthened the understanding of cellular signaling networks, regulatory pathways, and disease-associated molecular mechanisms [25]. Ferritin has been characterized for its chemical and biological properties, supporting its relevance as a biomolecule in biotechnology and biomedical research [26]. Nanobody-based systems have been explored as diagnostic and therapeutic tools because of their specificity, stability, and capacity for targeted molecular recognition [27]. Ferritin-based nanoplatforms have also been investigated for biological detection, imaging diagnosis, and drug delivery, demonstrating how nanoscale biomolecular systems can integrate multiple biomedical functions [28]. Research on fusion RNAs has improved understanding of oncogenic mechanisms and has potential diagnostic relevance in the molecular pathology of cancer [12]. Optical imaging-guided targeted sampling has been investigated as a method to improve precise diagnosis and molecular pathology assessment [14]. In computational pathology, standardized specimen and data provenance are important for AI development, diagnostic reproducibility, and safe clinical translation of algorithm-supported pathology workflows [19]. Together, these approaches show that biotechnology should be interpreted as a group of clinically established, emerging translational, and experimental technologies rather than as a single uniform category. Table 1 summarizes selected biotechnology-related techniques and their applications in medicine and pathology.

**Table 1:** Key biotechnological techniques and their applications in modern medicine. Created by the authors based on information synthesized from previously published studies [4,5,7,9,12-14,22,23,25-29]. Therefore, no copyright permission or license agreement was required. The techniques listed in this table differ in clinical maturity. Molecular diagnostics are established in many clinical workflows, whereas CRISPR-based stem cell manipulation, biomimetic nanoparticle platforms, ferritin-based nanoplatforms, nanobody-based systems, microbial biosensors, and photodynamic therapy remain primarily research or translational tools, with clinical applicability varying by disease context, platform validation, and implementation setting. CRISPR: clustered regularly interspaced short palindromic repeats

Biotechnological technique	Principle/Basis	Major application in medicine and pathology	Clinical/Research significance	References
Molecular diagnostics	Detects genetic, epigenetic, or molecular alterations using DNA-, RNA-, and biomarker-based assays	Detection and classification of genetic, infectious, and cancer-associated molecular alterations	Established in many clinical workflows, supports molecular disease classification and diagnostic interpretation	[7,9]
Fusion RNA and molecular pathology analysis	Evaluates disease-associated fusion RNAs and molecular alterations	Molecular pathology of cancer and investigation of oncogenic mechanisms	Supports molecular-level disease characterization and cancer-related diagnostic research	[12]
Optical imaging-guided targeted sampling	Uses imaging guidance to support targeted tissue sampling	Precise tissue sampling for diagnosis and molecular pathology assessment	May reduce sampling error and support localized diagnostic assessment	[14]
Microbial biosensors	Uses microbial systems to detect and respond to biological or environmental signals	Biosensing, signal detection, and biotechnology-based diagnostic development	Provides a biological platform for sensing and monitoring applications	[29]
Immuno-oncology	Uses immune-response modulation as a therapeutic strategy	Cancer treatment research and immune-mediated targeting of malignant disease	Represents a biotechnology-related therapeutic area in oncology	[22]
Biomimetic nanoparticle platforms	Uses biologically inspired nanoparticle systems for targeted delivery	Targeted delivery and treatment research using macrophage membrane-based nanoparticles	Supports therapeutic-delivery research and biomimetic platform development	[5]
CRISPR-based stem cell manipulation	Uses CRISPR methods in stem cell lines for experimental genetic modification	Stem cell research, disease modeling, and therapeutic-development studies	Primarily experimental and translational; supports stem cell-based research applications	[23]
Protein-protein interaction studies	Investigates interactions among proteins involved in signaling and regulation	Study of cellular signaling networks and disease-associated molecular mechanisms	Helps characterize molecular pathways relevant to health and disease	[25]
Ferritin-based nanoplatforms	Uses ferritin as a biomolecular platform for biomedical applications	Biological detection, imaging diagnosis, and drug delivery	Supports imaging, detection, and delivery research using biomolecular nanoplatforms	[26,28]
Nanobody-based systems	Uses engineered single-domain antibodies with high specificity and stability	Targeted diagnostic and therapeutic applications	Provides molecular-recognition tools for diagnostic and therapeutic research	[27]
In vitro culture systems	Uses controlled laboratory systems for biological growth and compound production	Secondary metabolite production and bioactive compound research	Supports controlled biomedical and pharmacological research applications	[13]
Photodynamic therapy	Uses light-activated molecular processes for therapeutic effects	Biotechnology-related therapeutic research	Represents a treatment-related biotechnology approach with biomedical relevance	[4]

Historical evolution of biotechnology in healthcare

The development of biotechnology in healthcare can be understood as a progressive shift from biological sensing and molecular characterization toward integrated diagnostic, therapeutic, and precision-medicine platforms. Early and foundational applications include microbial biosensors, which have been reviewed as biological systems capable of detecting and responding to specific signals, representing one biotechnology-based approach to sensing and diagnostic development [29]. Modern molecular diagnostic methods have expanded this foundation by enabling the detection and interpretation of disease-associated molecular alterations in clinical contexts [7]. Biomimetic repair materials, such as hydroxyapatite toothpaste, have also been evaluated for enamel remineralization and repair in deciduous teeth, illustrating specialty-specific applications of biotechnology in oral health [8].

The next major phase involved the expansion of nanomedicine, biomimetic delivery systems, and targeted therapeutic platforms. Nanomedicine has been discussed in coronary artery disease as a potential strategy for cardiovascular diagnosis and therapy [30]. Dental biotechnology has been reviewed in relation to prevention, diagnosis, and treatment in population oral health, illustrating how biotechnology-based strategies may support specialty-specific clinical applications beyond systemic medicine [31]. Multifunctional nanomedicine has also been investigated for targeted atherosclerosis therapy, including plaque clearance and inflammation-related mechanisms [10]. Nanotechnology has been reviewed for applications in cancer diagnosis and therapy [32], thrombus therapy [33], and the treatment and diagnosis of hypercholesterolemia [34]. Macrophage cell membrane-based nanoparticles have been investigated as biomimetic platforms for targeted delivery and treatment, demonstrating the use of biologically inspired engineering in therapeutic development [5]. Nanoscience-based conjugated drug strategies have also been discussed in relation to disease prevention and therapeutic development [35].

More recent biotechnology development has extended into regenerative, cellular, and mechanism-based therapeutic approaches. Mitochondria transfer and transplantation have been discussed as biotechnological approaches with therapeutic potential [6]. Photodynamic therapy has been reviewed as a biotechnology-related therapeutic approach with biomedical relevance [4]. Cellular disease-mechanism studies, including KLK8 upregulation in hippocampal neuronal apoptosis in a chronic unpredictable mild stress model, illustrate how biotechnology also supports mechanistic disease research [3]. These developments show that biotechnology has evolved from isolated laboratory techniques into a multi-domain field that now includes established diagnostic tools, translational nanomedicine platforms, and experimental regenerative or cellular interventions.

Molecular and cellular basis of modern biotechnology

Modern biotechnology is based on molecular recognition, cellular engineering, biomolecule characterization, nanoscale platform design, and computational integration of biological data. Microfluidics-based nanobiosensors have been reviewed as miniaturized systems for healthcare monitoring and biomarker-related analysis [36]. Ultrasound-based micro/nanosystems have been reviewed for biomedical applications, including imaging and therapy-related functions [37]. Photodynamic therapy has also been described as a biotechnology-related therapeutic approach involving light-activated molecular processes [4]. These platforms demonstrate how molecular and physical principles can be combined to support diagnosis, monitoring, imaging, and therapy.

At the cellular and molecular levels, biotechnology-related research includes stem cell maturation, biomolecule characterization, cytokine biology, and biomolecular nanoplatform development. Stem cell biotechnology includes challenges related to the in vitro maturation of hepatic stem cells and the development of functionally mature cell types [38]. Leukemia inhibitory factor has been reviewed in relation to biotechnology and cytokine-related biomedical implications [39]. Ferritin has been characterized in terms of its chemistry and biology, supporting its relevance as a biomolecule in biomedical research [26]. Ferritin-based nanoplatforms have been reviewed for biological detection, imaging diagnosis, and drug delivery [28].

Multi-omics and nanoscience have further expanded the molecular basis of biotechnology by enabling broader disease profiling and more targeted therapeutic design. Single-cell and spatial multi-omics approaches integrate genomic, transcriptomic, proteomic, epigenomic, metabolomic, and spatial information to support disease research, cellular heterogeneity analysis, tissue-context interpretation, and molecular stratification, although clinical translation remains limited by data complexity, computational integration, reproducibility, cost, and interpretability [40]. Nanoscience-based conjugated drug development has been discussed in relation to disease prevention and therapeutic applications [35]. Taken together, these molecular and cellular platforms provide the mechanistic foundation for precision medicine, but their clinical readiness differs substantially. Molecular diagnostics and selected biomarker assays are already used in clinical workflows, whereas many nanobiosensor, regenerative, and nanoplatform approaches remain translational or experimental.

Improvement in diagnostic techniques

Diagnostic development in biotechnology can be organized into four major domains: molecular diagnostics, minimally invasive diagnostics, biosensor and microfluidic platforms, and imaging- or nanotechnology-supported diagnostic systems. Modern molecular diagnostic methods have been reviewed as approaches for detecting molecular alterations relevant to clinical diagnosis [7]. Prokaryotic Argonautes have been investigated as molecular tools for in vivo biotechnology and molecular diagnostics [9]. RNA sequencing has been reviewed as a method for transcriptome-wide gene-expression analysis [41]. Molecular pathology has also been reviewed in the context of rare progeroid diseases, illustrating disease characterization at the molecular level [20]. Among these approaches, conventional molecular diagnostic assays are the most clinically established, whereas newer RNA-based and programmable molecular tools require disease-specific validation before routine implementation.

Minimally invasive and computational diagnostic approaches have strengthened disease monitoring and prognostic assessment. AI-based digital pathology methods have been evaluated for diagnostic accuracy, image-based pattern recognition, and pathology workflow support, although external validation and clinical integration remain important limitations [42]. Liquid biopsy approaches have been reviewed for minimally invasive cancer detection, molecular characterization, treatment-response monitoring, minimal residual disease assessment, and resistance surveillance, although performance remains influenced by assay sensitivity, tumor fraction, cancer type, disease stage, standardization, and clinical validation [43]. Tissue-specific drug delivery strategies have been reviewed in relation to ligand-, antibody-, cell-, and carrier-based targeting approaches, showing how molecular recognition systems may improve therapeutic localization and reduce off-target exposure [44]. Single-cell genomics has been discussed as a method for defining cell types and cell states [45]. These approaches may improve molecular stratification and longitudinal monitoring, but their diagnostic performance depends on disease type, sample quality, analytical platform, tumor burden, dataset representativeness, and external validation.

Biosensor, microfluidic, and nanotechnology-based platforms represent emerging diagnostic technologies with potential point-of-care relevance. Microfluidics-based nanobiosensors have been reviewed as small-volume analytical platforms for healthcare monitoring [36]. Microbial biosensors have been described as biological systems that can detect and respond to specific signals [29]. Ferritin-based nanoplatforms have been reviewed for imaging diagnosis, biological detection, and drug delivery, supporting their relevance in theranostic research [28]. Nanobody-based systems have been reviewed for diagnostic and therapeutic applications because of their specificity and stability [27]. These platforms offer potential advantages such as small sample requirements, portability, molecular specificity, and multifunctional diagnostic-therapeutic capacity; nevertheless, many remain primarily translational because sensitivity, specificity, reproducibility, scalability, and regulatory performance must be demonstrated in defined clinical settings.

Imaging-guided and nanoscale diagnostic systems can support lesion-directed assessment and integrated diagnostic-therapeutic strategies. Ultrasound-based micro- and nanosystems have been reviewed for biomedical imaging and therapy-related applications [37]. Nanotechnology has been reviewed in relation to thrombus therapy, including potential diagnostic and therapeutic applications [33]. Tissue-specific drug delivery strategies have been reviewed in relation to targeting approaches based on ligands, antibodies, cells, and carrier systems [44]. CRISPR manipulation in stem cell lines has been described as an experimental approach for genetic studies in stem cell research [23]. Therefore, diagnostic biotechnology should be interpreted according to clinical maturity: established molecular diagnostics support current clinical workflows, liquid biopsy and AI-assisted tools are clinically useful only in selected validated contexts, and many biosensor, nanoplatform, and CRISPR-related systems remain emerging or experimental. Table 2 lists the latest developments in diagnostic technologies based on biotechnology.

**Table 2:** Advances in diagnostic technologies in biotechnology. Created by the authors based on information synthesized from previously published studies [7,14,27,28,30,32,36,37,43]. Therefore, no copyright permission or license agreement was required. The technologies listed differ in clinical maturity, and performance metrics such as sensitivity, specificity, and AUC are assay-specific. These values should be reported only for validated tools in defined disease and platform contexts. AUC: area under the curve; POC: point-of-care

Diagnostic technology	Core working principle	Main diagnostic application	Clinical maturity/Validation status	Performance metrics or clinical advantage	References
Molecular diagnostic techniques	Detects DNA, RNA, mutations, fusion RNAs, and disease-associated molecular signatures	Diagnosis and molecular classification of cancer, inherited disorders, and infectious diseases	Established in many clinical laboratory workflows; performance varies by assay type and disease context	Assay-specific sensitivity, specificity, and turnaround time; no single universal diagnostic-performance value applies across all molecular diagnostic platforms	[7]
Nanoparticle-based diagnostics	Uses functionalized nanoscale systems to interact with disease-associated biomarkers or tissues	Cancer-related imaging, biomarker detection, and cardiovascular diagnostic or therapeutic research	Primarily research or translational; clinical maturity varies by nanoparticle type, disease indication, and validation status	Potential advantages include targeted signal generation, imaging support, and biomarker detection; disease-specific diagnostic accuracy values are not uniformly reported across platforms	[30,32]
Microfluidics-based biosensors	Integrates miniaturized fluid handling with biosensing platforms for small-volume sample analysis	Point-of-care testing, rapid screening, and biomarker monitoring	Primarily research and translational, with selected point-of-care applications depending on assay design and validation	Advantages include small sample volume, rapid analysis, portability, and potential cost reduction; performance is assay-specific	[36]
Optical imaging-guided diagnostic systems	Uses imaging-based visualization to guide targeted tissue sampling and molecular pathology assessment	Precise tissue sampling and lesion-directed diagnostic evaluation in pathology and oncology	Translational and procedure-dependent; requires validation for specific clinical indications	May reduce sampling error and improve localized diagnostic assessment; performance depends on imaging modality, target lesion, and clinical setting	[14]
Ultrasound-based micro/nanosystems	Combines ultrasound-responsive micro- or nanoscale agents with imaging platforms	Biomedical imaging and potential image-guided diagnostic or therapeutic applications	Primarily research or translational; clinical use depends on agent type, safety profile, and regulatory validation	Potential advantages include non-invasive imaging and integration with therapy-related functions; diagnostic-performance metrics vary by platform	[37]
Nanobody-based diagnostic systems	Employs engineered single-domain antibodies directed toward target antigens	Detection of disease-associated antigens and targeted molecular-recognition applications	Primarily research and translational, with diagnostic applicability depending on target, assay format, and validation	Advantages include stability and target specificity	[27]
Ferritin-based nanoplatforms	Uses ferritin nanocages as biomolecular carriers for detection, imaging, and delivery applications	Biomarker-related detection, imaging diagnosis, and theranostic research	Primarily preclinical or translational; not presented as a routinely established clinical diagnostic platform	Advantages include biocompatibility and multifunctional imaging/delivery potential	[28]
Liquid biopsy approaches	Detects circulating tumor DNA, circulating tumor cells, or other tumor-derived components in body fluids	Minimally invasive cancer detection, disease monitoring, and treatment-response assessment in genomics-driven oncology	Clinically used in selected oncology contexts, while broader applications remain under validation	Enables repeated sampling and molecular monitoring; diagnostic performance varies by cancer type, disease stage, analyte, and assay platform	[43]

Role of artificial intelligence and bioinformatics in diagnostics

AI and bioinformatics contribute to biotechnology-related diagnostics through data-driven analysis, molecular data integration, biomarker discovery, digital image interpretation, and predictive modeling in selected validated clinical contexts. Recent evidence from AI-based digital pathology shows that machine learning and related computational models may support diagnostic accuracy, image-based pattern recognition, biomarker quantification, and clinical workflow efficiency, although external validation, dataset heterogeneity, algorithmic transparency, and clinical integration remain important limitations [42]. Multi-omics approaches support the integration of genomic, transcriptomic, proteomic, and related datasets, providing a framework for systems-level analysis of disease biology [40]. Modern molecular diagnostic methods provide molecular-level information that can support disease classification and clinical interpretation when incorporated into diagnostic workflows [7]. Therefore, AI should be viewed as an assistive analytical layer rather than a replacement for molecular testing, histopathologic interpretation, or clinician-led decision-making.

Bioinformatics is particularly important for organizing and interpreting high-dimensional biological datasets. Multi-omics approaches require computational methods to integrate and interpret diverse molecular data types in disease research [40]. Liquid biopsy approaches provide minimally invasive molecular data, including circulating tumor-derived material, that may support disease monitoring and genomics-driven oncology workflows [43]. Machine learning methods may help identify diagnostic image patterns, support case triage, and assist biomarker-related assessment in digital pathology, although performance depends on dataset quality, model design, and validation strategy [42]. The main advantages of these tools include large-scale pattern recognition, biomarker discovery, and support for risk stratification. Their limitations include dataset heterogeneity, algorithmic opacity, variable reproducibility, and the need for external validation.

Computational pathology requires careful validation, data quality control, and clinically relevant interpretation. In computational pathology, standardized specimens and data provenance are important for AI model development, diagnostic reproducibility, and safe clinical translation [19]. Clinically, AI-supported pathology may assist with image-based pattern recognition, case triage, biomarker quantification, and prognostic modeling, but performance depends on representative training datasets, standardized workflows, explainability, and multicenter validation. Accordingly, AI and bioinformatics should be described as supportive analytical tools for molecular interpretation, prognosis prediction, and treatment-response assessment in selected validated contexts, rather than as universally established diagnostic replacements. Table 3 describes the integration of AI and bioinformatics in contemporary diagnostics and clinical implications.

**Table 3:** Role of artificial intelligence and bioinformatics in diagnostics. Created by the authors based on information synthesized from previously published studies [19,40,42]. Therefore, no copyright permission or license agreement was required. The tools listed differ in validation stage, and performance metrics such as accuracy, AUC, sensitivity, and specificity are model-, dataset-, and disease-specific. These values should be reported only for defined and validated tools rather than generalized across all AI or bioinformatics applications. AI: artificial intelligence; ML: machine learning; AUC: area under the curve

Tool/Approach	Functional basis	Diagnostic role	Validation stage/Evidence status	Performance metrics or clinical contribution	References
Bioinformatics for omics data analysis	Uses computational pipelines, algorithms, and databases to process genomic, transcriptomic, proteomic, metabolomic, and related datasets	Identifies biomarkers, disease-associated molecular patterns, and molecular alterations	Research and translational; clinical utility depends on disease context, data quality, and validation of analytical pipelines	Supports molecular stratification and systems-level disease interpretation; performance metrics are dataset- and platform-specific	[40]
ML algorithms in digital pathology	Learns patterns from biomedical datasets to classify disease features or predict outcomes	Image-based diagnostic support, pattern recognition, case triage, and biomarker-related assessment in pathology datasets	Research and translational; not presented as universally validated for routine diagnosis	Reported diagnostic accuracy varies by pathology task, dataset, model design, validation method, and external testing strategy; no universal AUC, sensitivity, or specificity applies across all AI pathology models	[42]
Predictive modeling in computational pathology	Applies AI-based or statistical models to integrate clinical and biological variables for outcome prediction	Supports diagnostic classification, risk stratification, or pathology-based prediction when validated in defined clinical datasets	Research and translational; external validation is required before clinical implementation	May support diagnostic classification, risk stratification, and pathology-based prediction when validated in defined clinical or pathology datasets	[42]
Computational pathology data provenance	Ensures traceability, quality control, and harmonization of pathology specimens and associated datasets	Supports validation and safe development of AI tools in computational pathology	Foundational requirement for AI development and clinical translation; not itself a diagnostic algorithm	Improves reliability, reproducibility, and regulatory readiness of computational pathology workflows	[19]
Standardized biomedical datasets	Uses curated, traceable, and harmonized datasets for algorithm development and evaluation	Supports training, testing, and validation of AI-based diagnostic or prognostic tools	Required for reproducible model development; validation depends on dataset representativeness and external testing	Supports transparent model evaluation; diagnostic metrics should be reported only for specific validated tools and datasets	[19]

Therapeutic innovations in biotechnology

Therapeutic innovations in biotechnology can be organized into immuno-oncology, nanomedicine and theranostics, targeted drug delivery, and mechanism-based therapeutic discovery. Immuno-oncology represents a clinically important biotechnology-related therapeutic area in which immune-response modulation has been applied to cancer treatment [22]. It is relatively more established for selected cancer indications, although response, toxicity, and survival benefit vary by tumor type, treatment class, and patient subgroup.

Nanomedicine has been reviewed for cancer diagnosis and therapy, including nanoscale systems designed for tumor imaging, drug delivery, and therapeutic targeting [32]. Ferritin-based nanoplatforms have been described as multifunctional systems for biological detection, imaging diagnosis, and drug delivery [28]. Nanotechnology has also been reviewed in thrombus therapy, where targeted delivery may address limitations of conventional thrombolytic strategies [33]. Nanoscience-based conjugated drug strategies have been discussed in relation to disease prevention and therapeutic development [35]. These approaches may improve tissue targeting and reduce off-target exposure, but most remain translational because safety, biodistribution, scalability, and clinical efficacy require further validation.

Targeted drug delivery strategies use ligands, antibodies, cellular carriers, and other molecular recognition systems to improve localization of therapeutic agents to selected tissues or cells [44]. Dental biotechnology also illustrates how biotechnology-based approaches may contribute to prevention and treatment within a defined clinical specialty [31]. Protein-protein interaction studies further support mechanism-based therapeutic discovery by identifying pathways relevant to health and disease [25]. Thus, therapeutic biotechnology should be interpreted by maturity level: selected immuno-oncology tools are clinically established, targeted delivery and nanomedicine platforms are emerging translational technologies, and many biomolecular or nanoscience-based systems remain experimental until supported by robust safety and efficacy data. Table 4 gives a review of biotechnological therapeutic innovations and how they work.

**Table 4:** Therapeutic innovations in biotechnology and mechanisms of action. Created by the authors based on information synthesized from previously published studies [1,5,16,22,23,28,30,32,33,44]. Therefore, no copyright permission or license agreement was required. Therapeutic approaches listed in this table differ in clinical maturity, and outcomes such as survival, response rate, and toxicity reduction are indication- and platform-specific. CRISPR: clustered regularly interspaced short palindromic repeats

Therapeutic innovation	Mechanism of action	Major clinical/Research application	Clinical maturity/Validation status	Therapeutic benefit/Outcome	References
Gene editing technologies	Uses CRISPR-based methods to modify genetic material in stem cell lines for experimental study	Stem cell research, disease modeling, and therapeutic-development studies	Experimental and translational; not presented as an established curative therapy	Supports genetic manipulation and disease-modeling research; clinical benefit depends on indication-specific validation	[23]
Targeted nanomedicine and tissue-specific drug delivery	Uses nanoparticles, ligands, antibodies, cellular carriers, or tissue-specific recognition elements to deliver therapeutic agents to selected tissues or cells	Autoimmunity, cancer-related nanomedicine, and tissue-specific therapeutic delivery	Research, translational, and indication-dependent; clinical maturity varies by platform and disease context	May improve local drug delivery and reduce off-target exposure where validated; survival, response, and toxicity outcomes are disease- and platform-specific	[16,44]
Cancer-focused nanomedicine	Uses nanoscale platforms for tumor imaging, drug delivery, and therapeutic targeting	Cancer diagnosis and therapy research	Primarily research and translational; clinical applicability varies by platform and cancer type	May support tumor imaging, targeted delivery, and therapeutic research, but outcome metrics are platform-specific	[32]
Cardiovascular and thrombus-related nanomedicine	Uses nanoscale systems for cardiovascular or thrombus-related diagnostic and therapeutic applications	Cardiovascular nanomedicine and thrombus therapy research	Primarily research and translational; validation depends on cardiovascular indication and platform type	May support targeted cardiovascular or thrombus-related delivery; safety and efficacy require indication-specific validation	[30,33]
Immuno-oncology	Uses immune-response modulation to target malignant disease	Cancer treatment research and selected oncology applications	Clinically established for selected cancer indications, while benefits vary by tumor type, treatment class, and patient subgroup	May expand treatment options and support durable responses in selected cancers; outcome metrics should be interpreted by disease context	[22]
Biomimetic nanocarriers	Mimics natural cell membranes or biological systems to support targeted delivery and biological compatibility	Targeted delivery and treatment research using macrophage cell membrane-based nanoparticles	Primarily research and translational	May support targeted delivery and biomimetic platform development; immune modulation and safety require careful evaluation	[5]
Theranostic nanoplatforms	Integrates biological detection, imaging diagnosis, and drug delivery functions within a nanoscale platform	Imaging diagnosis, biological detection, drug delivery, and theranostic research	Primarily preclinical or translational; not presented as routine clinical therapy	May support combined diagnostic and therapeutic research applications, but clinical utility requires further validation	[28]
Regenerative and stem cell-based approaches	Uses regenerative strategies, stem cell-related methods, or cell maturation approaches to support tissue repair and disease modeling	Endometrial regenerative therapy research and CRISPR-based stem cell-line manipulation	Research and translational; clinical applicability depends on tissue type, disease indication, and validation stage	May support tissue-repair research and disease modeling; long-term functional recovery should not be assumed without indication-specific evidence	[1,23]

Comparative clinical appraisal of biotechnology platforms

Biotechnology platforms differ substantially in clinical maturity, implementation readiness, and direct relevance to patient care. Established clinical tools, such as molecular diagnostics and selected immuno-oncology applications, already support disease classification, treatment selection, and clinical decision-making in defined settings [7,22]. Their main advantages include clinical familiarity, standardized workflows, and stronger evidence for diagnostic or therapeutic utility. However, their performance remains disease-specific and depends on assay quality, biomarker validity, patient selection, and local laboratory capacity [7].

Emerging translational technologies, including liquid biopsy, AI-assisted pathology, multi-omics integration, microfluidic biosensors, nanobody-based systems, and selected targeted delivery platforms, offer potential advantages such as minimally invasive monitoring, high-dimensional molecular profiling, improved risk stratification, and more precise therapeutic targeting [40,43]. Their broader clinical use remains limited by variable validation, dataset heterogeneity, reproducibility concerns, infrastructure requirements, and the need for prospective multicenter evidence [19,42].

Experimental approaches, including many regenerative, stem-cell, CRISPR-based, biomimetic nanocarrier, and theranostic nanoplatform systems, are mainly valuable for disease modeling, mechanistic research, and early therapeutic development [1,23]. These technologies may provide future clinical benefit, but they should not be interpreted as routine clinical tools until safety, efficacy, scalability, long-term outcomes, regulatory requirements, and cost-effectiveness are established [5,28]. Therefore, biotechnology should be evaluated through a maturity-based framework that distinguishes routine clinical implementation from translational promise and experimental feasibility.

Personalized and precision medicine

Personalized and precision medicine uses biotechnology to link molecular profiling, biomarker identification, targeted therapy, and longitudinal disease monitoring. Multi-omics approaches integrate genomic, transcriptomic, proteomic, metabolomic, and related datasets to support disease research and molecular stratification [40]. RNA sequencing provides transcriptome-wide assessment of gene-expression patterns and regulatory changes [41], while liquid biopsy enables minimally invasive detection and monitoring of circulating tumor-derived material in genomics-driven oncology [43]. Modern molecular diagnostic methods further support precision-oriented disease classification by detecting and interpreting clinically relevant molecular alterations [7].

Nanotechnology and targeted delivery systems are also central to precision-oriented therapeutic research. Macrophage cell membrane-based nanoparticles have been investigated as biomimetic platforms for targeted delivery and treatment [5]. Nanotechnology has been reviewed for cancer diagnosis and therapy, including tumor imaging, drug delivery, and therapeutic targeting [32]. Tissue-specific delivery strategies using ligands, antibodies, cellular carriers, and carrier-based systems are intended to improve localization of therapeutic agents and reduce off-target exposure where feasible [44]. Ferritin-based nanoplatforms have also been reviewed for biological detection, imaging diagnosis, and drug delivery, supporting their role in theranostic research [28].

**Figure 2:**
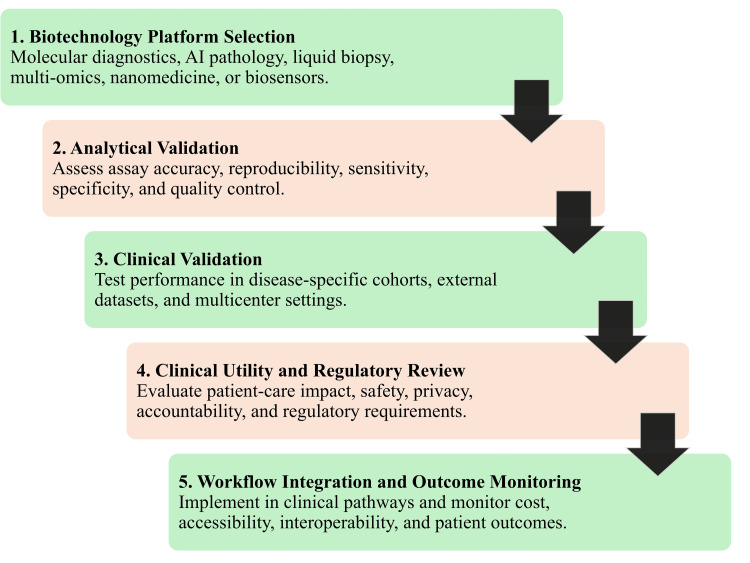
Clinical implementation pathway for biotechnology-enabled precision medicine. Created by the authors using Microsoft PowerPoint. Source: [40,41,43,44]. AI: artificial intelligence

These technologies differ in clinical maturity. Molecular diagnostics and selected liquid biopsy applications are already used in defined clinical contexts, whereas many multi-omics workflows, biosensor platforms, nanomedicine systems, and biomimetic delivery approaches remain translational or preclinical. Microfluidics-based nanobiosensors have been reviewed as small-volume platforms for healthcare monitoring and biomarker analysis [36], and biotechnology-based tissue delivery approaches, including chenodeoxycholic acid-related pharmaceutical applications, remain preclinical and require further evaluation before routine clinical use [21]. Thus, precision medicine should be viewed as an implementation pathway that requires validated biomarkers, reproducible assays, clinically interpretable data, and evidence that individualized treatment decisions improve patient outcomes. Figure 2 illustrates the stepwise pathway through which biotechnology platforms progress from selection and analytical validation to clinical validation, regulatory review, workflow integration, and patient-outcome monitoring.

Applications of biotechnology in pathology practice

Applications of biotechnology in pathology can be organized into molecular pathology, RNA-based analysis, nanotechnology-supported detection, and computational pathology. Molecular pathology focuses on disease-associated genetic, transcriptomic, and biomarker-related alterations. Research on fusion RNAs has contributed to understanding oncogenic mechanisms and has potential relevance for molecular pathology in cancer [12]. RNA sequencing has been reviewed as a transcriptome-wide method for assessing gene-expression patterns and regulatory changes, supporting molecular-level investigation of disease biology [41].

Nanotechnology-based platforms have also contributed to pathology-related diagnostic and therapeutic research. Ferritin-based nanoplatforms have been reviewed for biological detection, imaging diagnosis, and drug delivery [28]. Nanotechnology has been reviewed for cancer diagnosis and therapy, including nanoscale approaches to tumor imaging, drug delivery, and therapeutic targeting [32]. Nanobody-based systems have been reviewed for diagnostic and therapeutic applications because of their specificity, stability, and targeted molecular recognition properties [27]. Biomimetic multimodal nanoplatforms have also been investigated in cancer therapy research, including antiglioma applications [17]. These platforms may support imaging, biomarker detection, and targeted therapy research, but routine pathology implementation requires stronger validation, reproducibility, safety assessment, and clinical-utility evidence.

**Figure 3:**
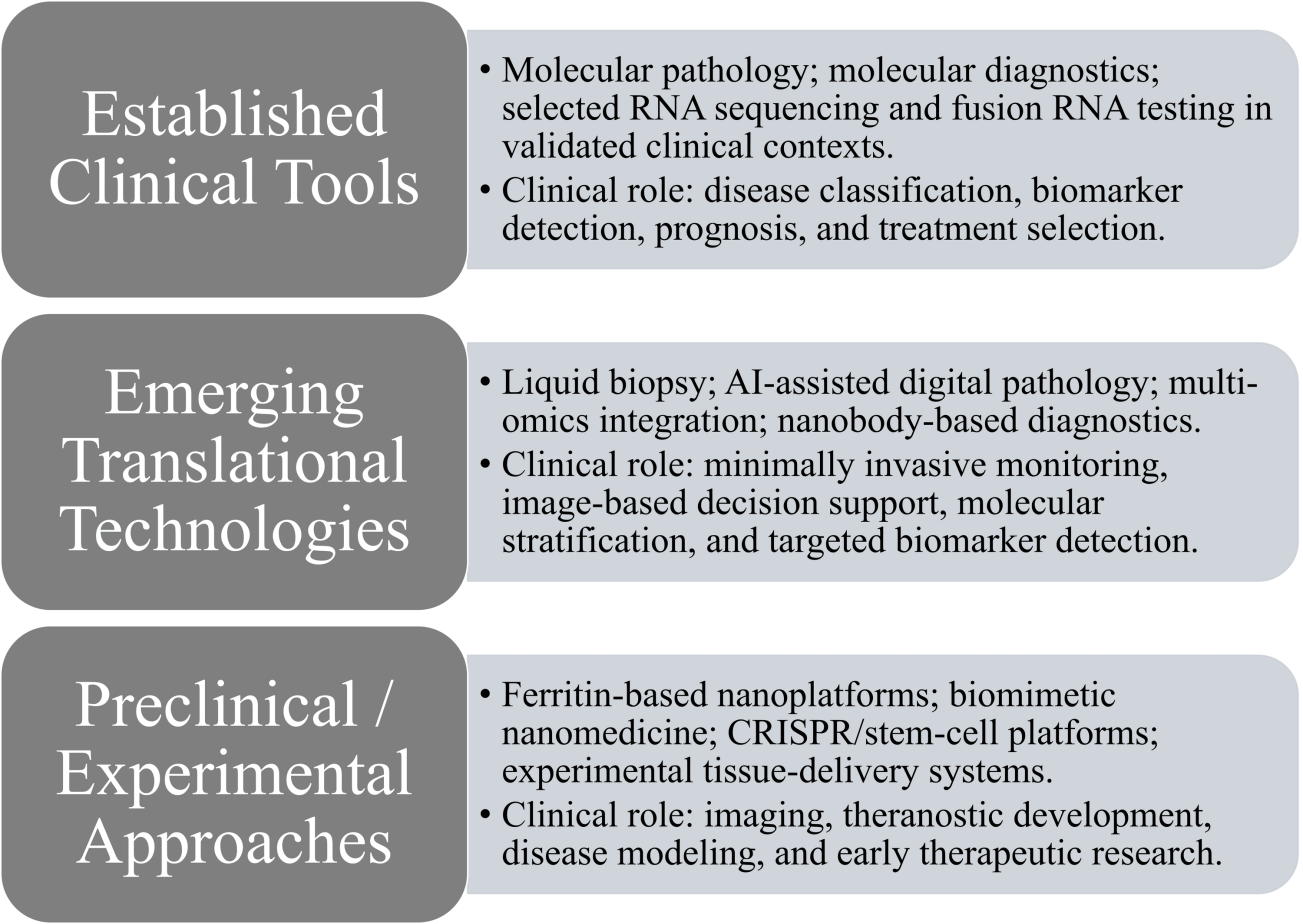
Comparative maturity framework for biotechnology applications in pathology. Created by the authors using Microsoft PowerPoint. Source: [7,12,14,19,27,28,36,43]. AI: artificial intelligence; CRISPR: clustered regularly interspaced short palindromic repeats

Digital and computational pathology should be interpreted as an assistive and validation-dependent domain rather than a replacement for a pathologist-led diagnosis. Specimen and data provenance are essential for developing reliable AI tools in computational pathology and for supporting reproducible interpretation of pathology data [19]. AI-based digital pathology methods have been evaluated for diagnostic accuracy and image-based decision support, indicating their relevance as assistive pathology tools rather than universal replacements for pathologist-led diagnosis [42]. Nanomedicine studies in acute pancreatitis and preclinical tissue delivery studies, including chenodeoxycholic acid-related pharmaceutical applications, further illustrate how disease-specific molecular mechanisms and delivery systems may be evaluated before clinical translation [24,21]. Accordingly, biotechnology-related pathology applications should be classified by maturity: molecular pathology is established in many clinical settings, AI-assisted pathology and nanobody-based diagnostics are emerging, and many nanomedicine or biomimetic delivery platforms remain preclinical or translational. Figure 3 categorizes biotechnology applications by clinical readiness, separating established tools, emerging translational technologies, and preclinical or experimental approaches.

Impact on clinical outcomes and patient care

The patient-care implications of biotechnology can be organized into improved diagnosis, targeted therapy, precision treatment selection, and disease monitoring. Modern molecular diagnostic methods support detection and interpretation of clinically relevant molecular alterations [7], while optical imaging-guided targeted sampling may improve lesion-directed diagnosis and molecular pathology assessment [14]. Nanotechnology has been reviewed for cancer diagnosis and therapy [32], coronary artery disease [30], and thrombus therapy [33], while tissue-specific delivery strategies aim to improve localization of therapeutic agents and reduce off-target exposure [44]. Immuno-oncology uses immune-response modulation to target malignant disease and represents a clinically important biotechnology-related cancer treatment field [22].

Clinical benefit depends on validation and maturity level. Established molecular diagnostics and selected immuno-oncology applications already influence care in defined settings, whereas regenerative therapies, ferritin-based theranostic nanoplatforms, preclinical tissue delivery systems, and many nanomedicine approaches remain translational or experimental [1,21,28]. Therefore, biotechnology may improve patient care through earlier detection, molecular stratification, targeted therapy, and treatment monitoring, but its clinical value depends on disease context, safety evidence, cost, accessibility, and implementation feasibility.

Limitations and future directions

The integration of biotechnology into medicine and pathology remains limited by cost, infrastructure requirements, technical expertise, validation gaps, regulatory complexity, and unequal access. Technologies such as genomic sequencing, nanomedicine, AI-assisted pathology, multi-omics analysis, and targeted therapies require standardized workflows, quality-control systems, trained personnel, and evidence of analytical validity, clinical validity, clinical utility, reproducibility, scalability, and long-term safety.

Data-related challenges include standardization, secure storage, interoperability, algorithmic transparency, bias, and interpretation of large-scale biological datasets. Ethical and regulatory concerns include data privacy, informed consent, equitable access, accountability for AI-assisted decisions, and post-implementation monitoring. Future work should prioritize low-cost point-of-care platforms, multicenter prospective validation, interoperable data standards, transparent algorithm reporting, cost-effectiveness evaluation, and regulatory pathways that clearly distinguish established clinical tools from emerging translational and experimental technologies.

## Conclusions

This review concludes that biotechnology has strengthened modern medicine and pathology through molecular disease characterization, biomarker-based diagnosis, targeted therapeutic development, digital pathology, AI-supported analysis, and precision-medicine workflows. Molecular diagnostics and selected immuno-oncology applications are already established in defined clinical settings, whereas many nanomedicine, multi-omics, biosensor, regenerative, and AI-assisted platforms remain translational or experimental and require stronger validation. Clinical implementation depends on analytical validity, clinical utility, safety, cost-effectiveness, infrastructure, ethical oversight, and equitable access. Continued development of accessible diagnostic platforms, safer therapeutic systems, standardized data practices, multicenter validation, and clear regulatory pathways may support broader adoption. Overall, biotechnology has substantial potential to improve diagnostic accuracy, therapeutic targeting, disease monitoring, and patient-centered care when applied according to evidence level and clinical maturity.
